# Direct real‐time intra‐operative imaging of human brain tumour vessels using intravital microscopy

**DOI:** 10.1002/ctm2.70084

**Published:** 2024-12-05

**Authors:** Diogo Moniz Garcia, Emmanuel Gabriel, Alfredo Quinones‐Hinojosa

**Affiliations:** ^1^ Department of Neurologic Surgery Mayo Clinic Florida Jacksonville Florida USA; ^2^ Department of Neurologic Surgery Washington University St. Louis Missouri USA; ^3^ Department of General Surgery, Division of Surgical Oncology Mayo Clinic Florida Jacksonville Florida USA

Dear Editor,

We report below on a novel intra‐operative use of intra‐vital microscopy for real‐time evaluation of microvessels in brain tumour patients, which enables the visualization of both functional and non‐functional vessels and the inspection of blood‐brain barrier (BBB) integrity.

Brain cancer's prognosis remains dismal despite significant strides in our collective understanding of its biology and numerous efforts towards developing new strategies to target its main growth and resistance engines.^1^, ^2^ This can be partly explained by the presence of the BBB,^3^ which significantly limits the ability of drugs to penetrate the brain. While some regions of BBB disruption can be found in brain cancers, areas of intact BBB can be found even in the most aggressive of brain cancers, glioblastoma.^4^, ^5^ Therefore, numerous strategies have been developed aimed at producing more brain‐penetrant drugs with mixed results. This has motivated intense research into developing novel technologies to open the BBB to enable more effective drug delivery, such as focused ultrasound.^6^, ^7^ However, the ability to visualize the effect of these enabling technologies in real‐time in the operating room has remained elusive thus far. We optimized an emerging technology, employing human intravital microscopy (HIVM) to analyze microvasculature in the non‐infiltrated and tumour‐infiltrated brain in real‐time in the operating room during already occurring brain tumour surgeries. This allowed the observation of areas of intact BBB and areas of compromised BBB where fluorescein leaked into the brain parenchyma.

We performed a single centre, non‐randomized, prospective case series of real‐time HIVM observation of grossly non‐infiltrated cerebrum, cerebellum and brain tumours at the Mayo Clinic, Jacksonville between November 2023 and May 2024 (IRB 18–010370 under the leadership of AQH, who performed the brain surgeries for patients enrolled in the study). Inclusion criteria included patients undergoing open craniotomies for brain tumours, provided that allergic reactions to fluorescein were excluded. Biopsies, both stereotactic and open as well as infection and traumatic cases were excluded. The feasibility of intra‐operative use of HIVM had been established by our group in previous reports of other cancer types, including ovarian cancer and melanoma.^8^, ^9^ To enable real‐time visualization, fluorescent dye fluorescein (AK‐FLUOR) was administered intravenously to patients after exposure to the pertinent brain anatomy to enhance microvasculature imaging. Importantly, before administration of fluorescein, prick tests were performed in every patient to exclude allergic reactions, with any sign of allergic reaction representing an immediate exclusion criterion from further analysis or administration of fluorescein (no patients in the present study showed any allergic reaction to prick testing). After the administration of fluorescein, we proceeded to deploy an ultra‐high definition (UHD) probe associated with a confocal laser endomicroscopy device (pCLE; Gastroflex, Cellvizio System, Mauna Kea Technologies) was used under the neurosurgeon guidance to allow for observations at 100x magnification of the microvasculature in at least 2 non‐contiguous regions of brain tumours and 2 non‐contiguous regions of grossly normal brain. Analysis of the microvasculature was then performed using the Mauna Kea Technologies IC‐Viewer.^8^ In total, HIVM was performed in 10 patients (three males and seven females, average age of 53.7 years) with different histological subtypes of brain tumours (Table 1 and Figure 1). Functional vessels in grossly non‐infiltrated areas were found to have larger diameters and flow velocity (mean ± SD) when compared with those within tumour areas in matched patients (27.8 ± 30.1μ m vs.19.0 ± 29.5μ m, *p =* 0.0013 and 193.8 ± 61.8μ m/s vs. 94.2 ± 59.1μ m/s, *p =* 0.0001, respectively). Given the potential therapeutic impact of modulating the microvasculature in gliomas,^6^ an analysis focusing on glioma patients was pursued. Indeed, the same was observed (32.3 ± 34.4μ m vs.19.1 ± 30.2μ m, *p = *0.0024 and 171.9 ± 47.5μ m/s vs. 92.3 ± 63.4μ m/s, *p = *0.0001 for diameters and velocity, respectively). In line with our previous findings in other cancers,^8^, ^9^ we also observed numerous non‐functioning vessels (i.e., vessels that did not support blood flow), accounting for up to 50 % of all vessels evaluated in glioblastomas. Interestingly, despite the limited sample size, we observed that all vessels analyzed in the oligodendroglioma patient were functioning, which was higher than the proportion observed in other glioma types (100% vs. 66.7%, *p *< 0.0001).

**TABLE 1:** Characteristics of patients undergoing brain tumour surgery with intra‐operative human intravital microscopy (HIVM) analysis.

				FV diameter—mean (SD)^a^		Flow velocity—mean (SD)^a^	
ID	Age, Gender	Pathology	WHO	Control	Tumour	Control	Tumour
Patient 1	60, F	GBM	IV	44.3 (39.3)	13.3 (7.2)	148.1 (23.5)	64.3 (9.7)
Patient 2	39, M	Astrocytoma	II	37.4 (54.5)	18 (5.2)	99.6 (15)	22.1 (2.6)
Patient 3	73, F	GBM	IV	25.8 (23.5)	13.5 (11.6)	182.2 (28.4)	77.3 (11.2)
Patient 4	35, M	Astrocytoma	II	41.5 (35.3)	5.9 (1.4)	229.3 (36.3)	66.9 (44)
Patient 5	30, M	Oligodendroglioma	III	34 (33.9)	24 (39.2)	166.2 (57)	225.9 (44)
Patient 6	83, F	Meningioma	II	14.4 (17.2)	n.a.	230 (40.8)	n.a.
Patient 7	30, F	Metastatic Melanoma	n.a.	24.7 (20.2)	26.6 (17)	189.6 (40.5)	66.9 (15.7)
Patient 8	60, F	GBM	IV	30.2 (26)	38.1 (28.3)	203.3 (31.5)	105.4 (18.6)
Patient 9	55, F	Meningioma	I	26 (21.6)	9.4 (2.9)	315.1 (20.3)	135 (36.2)
Patient 10	72, F	GBM	IV	12.6 (4.5)	9.6 (0.4)	174.5 (11.1)	84 (22.5)

*Note*: WHO: World Health Organization; FV: Functional vessels; SD: Standard deviation; GBM: Glioblastoma; n.a.: not applicable; F: Female; M: Male.

^a^ The average functional vessel diameter was observed to be significantly higher in non‐infiltrated tissues at a mean (SD) diameter of 27.8μ m (30.1), when compared with brain tumours 19.0 μ m (29.5) **(*p =*** **0.0013)**, with the same result being observed when restricting to only gliomas. Flow velocity was also found to be significantly higher in non‐infiltrated tissues at an average of 193.8 μ m/s (61.8) when compared with brain tumour tissues at 94.2 μ m/s (59.1) **(*p =*** **0.0001)**. The same results were also observed when restricting to only glioma cases.

**FIGURE 1:**
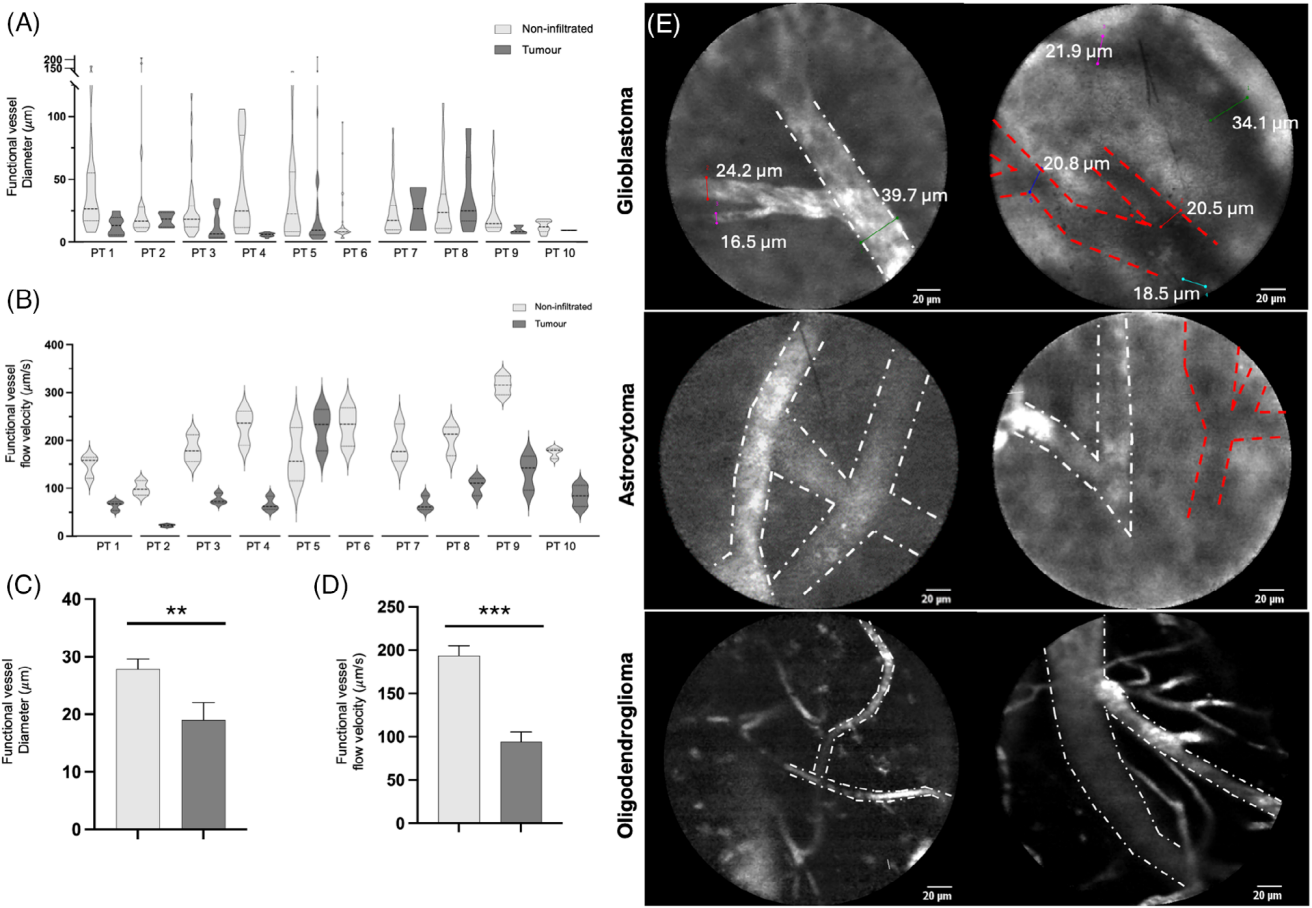
Human intravital microscopy (HIVM) in brain tumours. (A) Assessment of the diameter in μ m of functional vessels in included patients with a comparison between non‐infiltrated and infiltrated tumour areas from the same patients; (B) flow velocity of functional vessels comparing infiltrated and non‐infiltrated areas in μ m/s; diameter (C) and flow velocity of functional vessels (D) were found to be significantly higher in non‐infiltrated areas; (E) representative HIVMs from included patients in glioblastomas, astrocytomas and oligodendrogliomas demonstrating functional vessels (white dashed line) and non‐functional vessels (red dashed line). (C, D) *p < .05, **p < .01, ***p < .001 (unpaired student t‐test). *n* = 10. Data is mean ± s.e.m.; PT = Patient.

## 1 CONCLUSION

Our results show that HIVM enables the intra‐operative evaluation of both anatomy and blood flow dynamics of the microvasculature of both non‐infiltrated and infiltrated brain tissues in real‐time. Our experience shows that HIVM can detect areas of BBB integrity in glioblastomas, with clear flow through functioning vessels without noticeable leakage into the parenchyma, as well as areas of clear BBB disruption and leakage,^4^, ^5^ in a direct demonstration of what was previously reported from magnetic resonance imaging and positron emission tomography studies. Further, as previously demonstrated in other cancer models,^8^, ^9^ we observed that non‐functioning vessels are a significant proportion of all tumour microvessels particularly in the most aggressive type of gliomas, glioblastomas. Interestingly, in our series, in the oligodendroglioma case, we only observed functional vessels, which underline its different biological background^10^ and the need for further studies into its unique development.

In summary, this report demonstrates the feasibility of using HIVM in the Operating room (OR) during already occurring procedures to assess the integrity of the BBB in real‐time as well as vessel anatomical and dynamic assessment. This, in turn, will allow future studies aimed at demonstrating the efficacy and feasibility of BBB opening with enabling technologies.

## AUTHOR CONTRIBUTIONS

**Diogo Moniz Garcia and Emmanuel Gabriel**: Performed data collection and data analysis; **Diogo Moniz Garcia**: Performed original writing; **Emmanuel Gabriel and Alfredo Quinones‐Hinojosa**: Performed writing editing and supervision.

## CONFLICT OF INTEREST STATEMENT

The authors declare no conflict of interest

## FUNDING INFORMATION

Alfredo Quinones‐Hinojosa would like to ackowledge the support of Richard and Lauralee Uihlein, the William J. and Charles H. Mayo Professorship, the Mayo Clinic Clinician Investigator Award, the Florida Department of Health Cancer Research Chair Fund, and the Monica Flynn Jacoby Endowed Chair.

## ETHICS STATEMENT

All research reported in the current manuscript was approved by the institutional review board 18–010370.

## Data Availability

Data will be made available upon request and approval by the IRB of the performing institution.
